# The role of interleukin-13 in the removal of hyper-radiosensitivity by priming irradiation

**DOI:** 10.1093/jrr/rru053

**Published:** 2014-06-25

**Authors:** Nina F. Jeppesen Edin

**Affiliations:** Department of Physics, Biophysics Group, University of Oslo, PB 1048, Blindern, N-0316 Oslo, Norway

**Keywords:** interleukin-13, low-dose hyper-radiosensitivity, IL-13Rα2, iNOS, proprotein convertase

## Abstract

It has previously been demonstrated that the presence of fetal bovine serum is necessary for TGF-β3 (transforming growth factor beta 3)-dependent elimination of low-dose hyper-radiosensitivity (HRS) in cells by 1 h of low-dose-rate γ-irradiation (0.2–0.3 Gy/h). The purpose of the present study was to identify the serum constituent involved. Two human HRS-positive (T-47D, T98G) cell lines were used. The effects of different pretreatments on HRS were investigated using the colony assay. Total inducible nitric oxide synthase (iNOS) levels were measured using a cell-based ELISA assay. The serum factor was identified as interleukin-13 (IL-13). In order for low dose-rate irradiation to eliminate HRS through the TGF-β3-dependent mechanism, the cells must be exposed to IL-13 first. Inhibiting receptor IL-13Rα2 showed that this receptor is involved in the response. Adding IL-13 to serum-free medium restored the properties of full medium but not when an inhibitor of proprotein convertase activity was added together with IL-13. The presence of IL-13 resulted in upregulation of total iNOS protein levels. Thus, this study indicates that IL-13 interacts with the cells though receptor IL-13Rα2 and induces upregulation of iNOS and activation of one or more furin-like proprotein convertases.

## INTRODUCTION

Low-dose hyper-radiosensitivity (HRS) is, in all probability, the default response of cells to small doses of both high- and low-LET (linear energy transfer) ionizing radiations [1]. HRS is characterized by a high sensitivity per unit dose for doses below ∼0.5 Gy. For low-LET radiation, this is followed by a more resistant response in the dose range ∼0.5–1 Gy, called ‘increased radioresistance’ (IRR) [2, 3].

HRS is transiently removed in response to a high-dose-rate (HDR) priming dose of 0.2–0.3 Gy [4–8]. However, previous studies from our laboratory have shown that by protracting the delivery of a priming dose of 0.06–0.3 Gy to 1 h (LDR), the effect on HRS in T-47D breast cancer cells and T98G glioblastoma cells becomes permanent [4, 9]. The effect of LDR irradiation was found to depend on TGF-β3 (transforming growth factor beta 3), which turned off the HRS response in the cells [9]. In cells receiving medium from LDR-irradiated cells, as well as in cells receiving LDR-irradiated cell conditioned medium (medium conditioned on unirradiated cells, filtered and irradiated without cells present) or medium supplemented with recombinant TGF-β3, the effect on the HRS was transient. However, in cells that were LDR irradiated themselves, the HRS-response was removed permanently. Both the mechanism that induced the non-HRS response and the mechanism maintaining the non-HRS response were found to depend on iNOS activation and peroxynitrite generation from nitric oxide (NO) and superoxide [9, 10].

LDR irradiation had no effect on HRS if the cells were cultured in medium without fetal bovine serum. Also, the removal of HRS in cells receiving LDR-irradiated cell conditioned medium depended on the presence of serum during conditioning of the medium, but not during LDR irradiation [10].

Interleukin-13 (IL-13) is a pleiotropic immune regulatory cytokine with many characteristics in common with interleukin-4 (IL-4). The effect of both these cytokines is mediated through a cell surface receptor system consisting of IL-4 receptor α (IL−4Rα) and IL-13Rα1, which induces signal transduction through the Janus-activated kinase (JAK)/signal transducer and activator of transcription 6 (STAT6) pathway [11, 12]. IL-13 also binds to IL-13Rα2 with high affinity. This receptor was long thought to be a decoy receptor, with its main function being sequestering IL-13 to prevent binding to IL-13Rα1 [13]. However, IL-13Rα2 has been found to activate an AP-1 variant containing c-jun and Fra-2 [14], to induce iNOS (inducible nitric oxide synthase) upregulation [15, 16] and to be involved in the conversion of pro-furin to active furin [17]. Mandal and Levine found that whether IL-13Rα2 functions as an inhibitory decoy receptor depends on its relative expression being high compared with IL-13Rα1, and that cells with low levels of the receptor respond to IL-13 by biasing the cell toward enhanced MAPK signaling [18].

The aims of the present study were to find the putative factor in fetal bovine serum requisite for LDR irradiation to have an effect.

## MATERIALS AND METHODS

### Cell culture

Cells of two human HRS-positive cell lines (T-47D breast cancer cells and T98G glioblastoma cells) were used. The culture conditions were as described previously [4, 10, 19] using RPMI1640 (Roswell Park Memorial Institute) medium (JRH Biosciences, Kansas, USA) supplemented with 10% fetal calf serum (Gibco, Paisley, UK), 2 mM L-glutamine (SIGMA, St Louis, MO*,* USA), 200 units l^−1^ insulin (SIGMA), and 1% C. The cells were kept in exponential growth by reculturing of stock cultures two times a week. The cells were tested negative for the presence of mycoplasma.

In experiments with interleukins 4 or 13 (IL4–10H and IL13–22H, Creative Biomart, NY 11967, USA), the cells were washed three times with serum-free medium before serum-free medium with 10 ng/ml interleukin was added for cell conditioning and LDR priming. Fetal calf serum (10%) was added 1 h after the end of irradiation.

Decanoyl-RVKR-CMK (Tocris Bioscience, Bristol, BS11 0QL, UK) (20 μM) was used to block the activity of all seven proprotein convertases (PC1, PC2, PC4, PACE4, PC5, PC7 and furin). It was added together with 10 ng/ml IL-13 in serum-free medium to the cells 4 h before LDR priming. The medium was exchanged with normal full medium 1 h after the end of irradiation.

IL-13, IL-13Rα2 and TGF-β3 neutralizing antibodies were purchased from R&D (R&D systems, Minneapolis, MN, USA).

### Irradiation procedures

The cells were irradiated as described previously (19). The high dose rate (HDR) was ∼ 30 Gy/h as in previous reports, but because of [^60^Co]-decay, the low dose rate (LDR) used in the present experiments was ∼0.2 Gy/h (compared with 0.3 Gy/h in our previous studies). The total LDR irradiation time was 1 h, so the total dose in all LDR radiations was ∼0.2 Gy.

### Transfer of cell*-*conditioned medium irradiated without cells present

The medium transfer experiments in Fig. 2 were performed as described previously [10, 19] and summarized in Fig. 2A.

### Cell survival

The surviving fraction was determined by counting the number of colonies containing more than 50 cells as described previously [19].

### Statistical analysis

All experiments were repeated at least three times, using five flasks for each challenge dose and 10 for controls. Within each experiment, the arithmetic means were calculated, weighing the errors. Radiation survival curve data were fitted using the linear quadratic (LQ) or the induced repair (IR) model [4, 10, 19].

The two-tailed Student's *t*-test was used to compare the surviving fractions in response to the HDR challenge irradiation of pretreated cells compared with the controls or LDR-irradiated cells.

### Cell-based ELISA

The total iNOS levels in whole cells were measured by an ELISA-based assay using fluorogenic substrates according to the manufacturer's protocol (KCB9502, R&D systems, Minneapolis, MN, USA). Cells were grown overnight in microplates with or without IL-13 antibody (3 μg/ml) and fixed and permeabilized with 4% formaldehyde. The cells were then incubated simultaneously with two primary antibodies (iNOS and GAPDH for normalization) and thereafter with two secondary antibodies (horseradish-peroxidase and alkaline phosphatase). The fluorescence was measured using a Synergy 2 Multi-Mode Microplate Reader (BioTek, Winooski, VT, USA), and the fluorescence of iNOS was normalized to that of GAPDH for each well after background subtraction.

## RESULTS

**Fig. 1.**
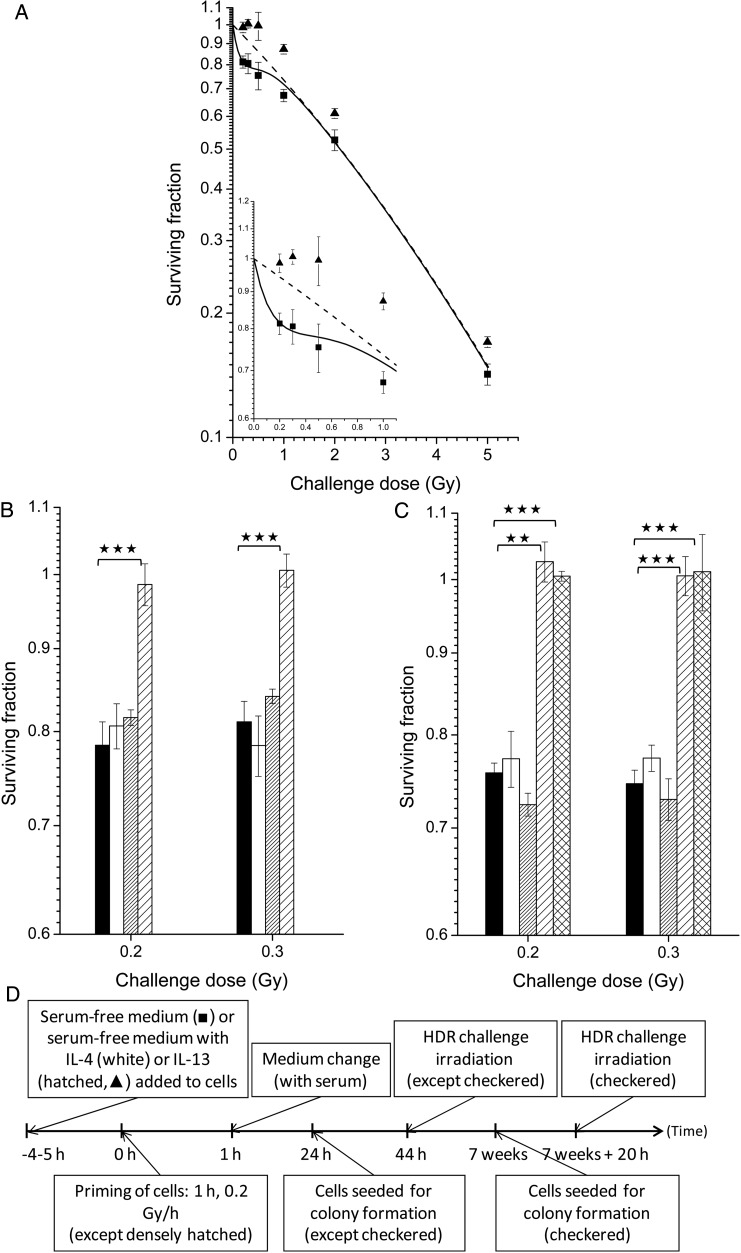
The effect on radiation response of pretreatments using interleukins in serum-free medium with or without LDR priming irradiation. The response was measured by surviving fractions as a function of HDR challenge dose (∼30 Gy/h). **(A)** LDR priming irradiation of T-47D cells with serum-free medium with (closed triangles) or without (closed squares) addition of 10 ng/ml IL-13, 40 h before HDR challenge irradiation (*P* < 0.01 for 0.2, 0.3 and 2 Gy, *P* = 0.02 for 5 Gy). The curves represent model-fits to the data from unprimed cells by the IR-model (solid lines) and the LQ-model (dashed lines), respectively. The bars represent standard errors of the mean (SEM) for three to seven individual experiments. T-47D **(B)** and T98G **(C)** cells pretreated with LDR priming irradiation of cells with serum-free medium supplemented with 10 ng/ml IL-4 (white), serum-free medium supplemented with 10 ng/ml IL-13 but no priming irradiation (densely hatched), LDR priming irradiation of cells with serum-free medium with 10 ng/ml IL-13, 24 h before HDR challenge irradiation (sparsely hatched), LDR priming irradiation of cells with serum-free medium with 10 ng/ml IL-13, 7 weeks before HDR challenge irradiation (checkered). Data from untreated cells are shown as reference (black). Two asterisks indicate *P* < 0.01; three asterisks indicate *P* < 0.001. The bars represent SEM for three individual experiments. **(D)** Timeline showing the experimental schedules.

From previous experiments, it was known that the presence of serum was essential for LDR irradiation to have an effect on HRS [10]. In order to identify the factor required in the serum, interleukins 4 and 13 were tested on both T-47D cells (Fig. 1A and B) and T98G cells (Fig. 1C). In Fig. 1A, the full dose–response curve of the HRS region is shown for cells that were LDR-primed with serum-free medium with or without IL-13. It indicates that IL-13 can replace serum for the effect of LDR priming to HRS. In Fig. 1B and C, the two doses within the HRS range with the largest difference in survival of HRS-positive and HRS-negative cells, 0.2 and 0.3 Gy, were used as challenge doses. IL-13 in serum-free medium did not influence radiosensitivity itself. However, the presence of IL-13 in serum-free medium was sufficient to make LDR irradiation induce resistance to HDR challenge doses of 0.2 and 0.3 Gy, even 7 weeks after priming irradiation. LDR irradiation of cells with serum-free medium with IL-4 did not influence radiosensitivity.

**Fig. 2.**
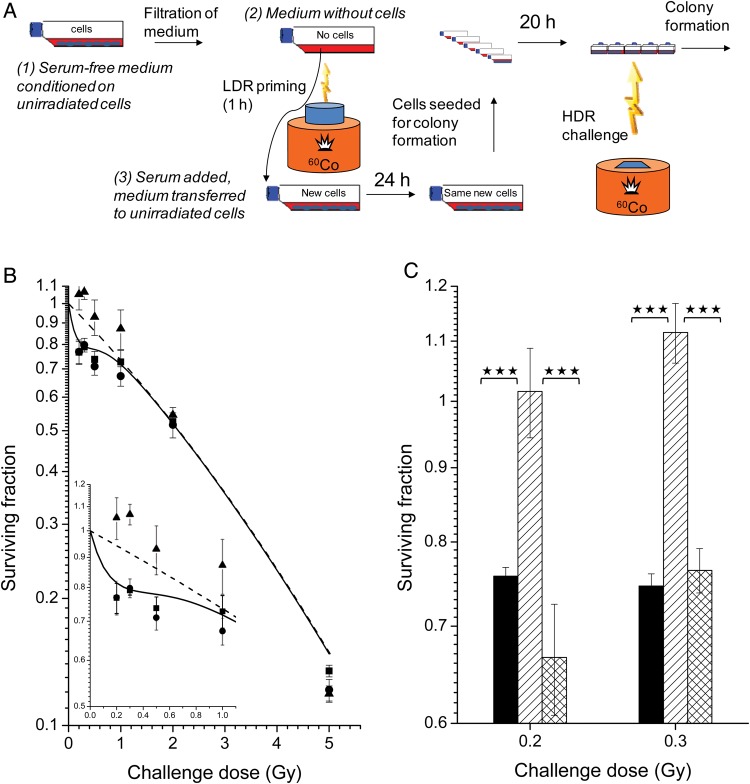
The response to HDR challenge irradiation of cells receiving medium that was conditioned without serum on unirradiated cells in the presence of IL-13 and subsequently priming irradiated without cells present. **(A)** Experimental design: serum-free medium was first conditioned on unirradiated cells (Step 1). It was then removed from the cells, filtered (0.22 μm) and LDR irradiated (0.2 Gy/h) for 1 h (Step 2). Immediately following irradiation, the medium was supplied with 10% serum and added to untreated (recipient) cells (Step 3). The recipient cells were exposed to the transferred medium for 24 h before being seeded for colony formation in fresh medium for 16–20 h before challenge irradiation. For the experiments shown in the checkered bars in Fig. 2C, the recipient cells were given medium exchange after being exposed to the transferred medium for 24 h. They were then cultured for 2 weeks (with 2-weekly re-seedings) before being seeded for colony formation and challenge irradiation. **(B)** T47D cells: serum-free medium, which had been cell conditioned (Step 1 in panel A) with (closed triangles) or without (closed squares) 10 ng/ml IL-13 for 24 h, and LDR irradiated for 1 h (Step 2 in panel A) with (closed circles) or without 2 μg/ml TGF-β3 neutralizer (*P* < 0.01 for 0.2 and 0.3 Gy, *P* < 0.05 for 0.5 Gy comparing cells receiving irradiated medium first conditioned with (closed circles) or without (closed triangles) IL-13; *P* < 0.01 for 0.3 Gy, *P* < 0.05 for 0.2 and 0.5 Gy comparing cells receiving medium conditioned with IL-13 and irradiated with (closed circles) or without (closed triangles) TGF-β3 neutralizer. The curves represent model-fits to the data from unprimed cells by the IR-model (solid lines) and the LQ-model (dashed lines), respectively. The bars represent standard errors of the mean (SEM) for three individual experiments. **(C)** T98G cells: serum-free medium, that had been cell conditioned with 10 ng/ml IL-13 (shown as closed triangles in panel B). The recipient cells were seeded for colony formation and challenge irradiation, either directly after 24-h exposure to the transferred medium, as shown in panel A (hatched), or 2 weeks after (checkered) (see details under **(A)**). Data from untreated cells are shown as reference (black). Three asterisks indicate *P* < 0.001. The bars represent SEM for three individual experiments.

Figure 2 shows data from exposing cells to medium, which had first been conditioned without serum on unirradiated cells and then LDR irradiated without serum or cells present (experimental design is shown in Fig. 2A). A previous study [10] showed that when full medium was used, this resulted in elimination of HRS in recipient cells. As seen in Fig. 2B, cells receiving medium that was cell conditioned and LDR irradiated without serum present, retained the HRS response. However, when the experiment was repeated with IL-13 added to the serum-free medium during cell conditioning (and priming), the recipient cells lost the HRS response when tested 40 h after the end of exposure to the transferred medium. Contrary to when the cells (and not only cell-conditioned medium) were LDR primed (Fig. 1 B and C (checkered)), the effect did not last and HRS was regained when tested 2 weeks later (Fig. 2B (checkered)). This is similar to observations using cell-conditioned serum-containing full medium [10].

We have previously shown that TGF-β3 is involved in the response to LDR irradiation [20]. The presence of a TGF-β3 neutralizer inhibited the effect of LDR-priming irradiation, and the cytoplasmic levels of TGF-β3 were found to be significantly higher in LDR-primed cells than in unprimed control cells. In addition, recombinant TGF-β3 was found to remove HRS in unprimed cells [20]. Since IL-13 without LDR irradiation did not affect the HRS-response (Fig. 1B and C), it is possible that IL-13 is involved in proactivation of the TGF-β3-dependent mechanism before final activation by LDR irradiation. Concentrations as low as 0.001 ng/ml TGF-β3 have previously been found to remove HRS [20]. However, due to the sensitivity of the ELISA assay for TGF-β3, only concentrations at least 100 times higher could be measured by this method [20]. We therefore had to use a more indirect method to test the involvement of IL-13 in TGF-β3 activation and added TGF-β3–neutralizer to cell-conditioned serum-free medium with IL-13 before LDR irradiation (Step 2 in Fig. 2A). This prevented the removal of HRS in the recipient cells.

**Fig. 3.**
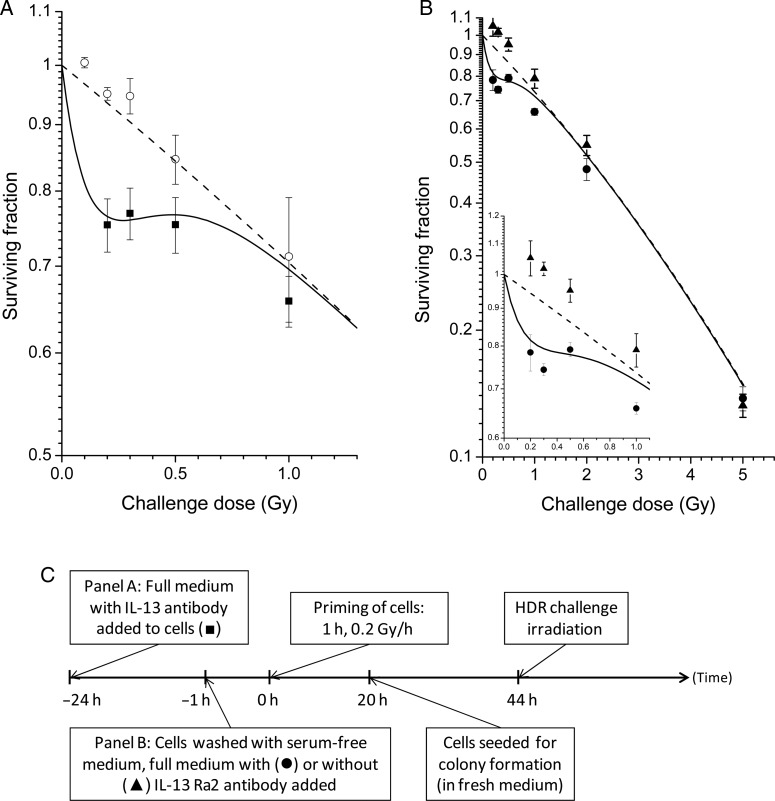
The response to HDR challenge irradiation in cells LDR primed during inhibition of IL-13 or receptor IL-13Rα2. **(A)** Full medium with 3 μg/ml IL-13-neutralizing antibody was added to T98G cells 24 h prior to LDR priming irradiation (closed squares). Data for LDR-primed cells without antibody are redrawn from a previous study [9] (open circles) (*P* = 0.05 for 0.2 Gy, *P* = 0.02 for 0.3 Gy, comparing cells LDR primed with or without IL-13-neutralizing antibody). **(B)** T-47D cells were washed three times in serum-free medium and then LDR primed after transfer of full medium with (closed circles) or without (closed triangles) receptor IL-13Rα2 neutralizing antibody (*P* < 0.001 for 0.3 Gy, *P* < 0.05 for 0.2 and 0.5 Gy). The curves represent model-fits to the data from unprimed cells by the IR-model (solid lines) and the LQ-model (dashed lines), respectively. The bars represent standard errors of the mean (SEM) for three individual experiments. **(C)** Timeline showing the experimental schedules.

In order to test whether IL-13 was the only constituent of serum with the observed effect, a neutralizing antibody was added the day before LDR priming of cells with full medium (Fig. 3A). These cells did not lose the HRS response, confirming that IL-13 is indeed solely responsible for the effect.

Since IL-13 and not IL-4 was able to influence to ability of the cells and medium to respond to LDR irradiation, it was hypothesized that the effect was mediated by receptor IL-13Rα2, which is specific for IL-13. This was confirmed by inhibiting the receptor using an IL-13Rα2-antibody 1 h before and during LDR priming, which disrupted the effect of the LDR priming (Fig. 3B).

**Fig. 4.**
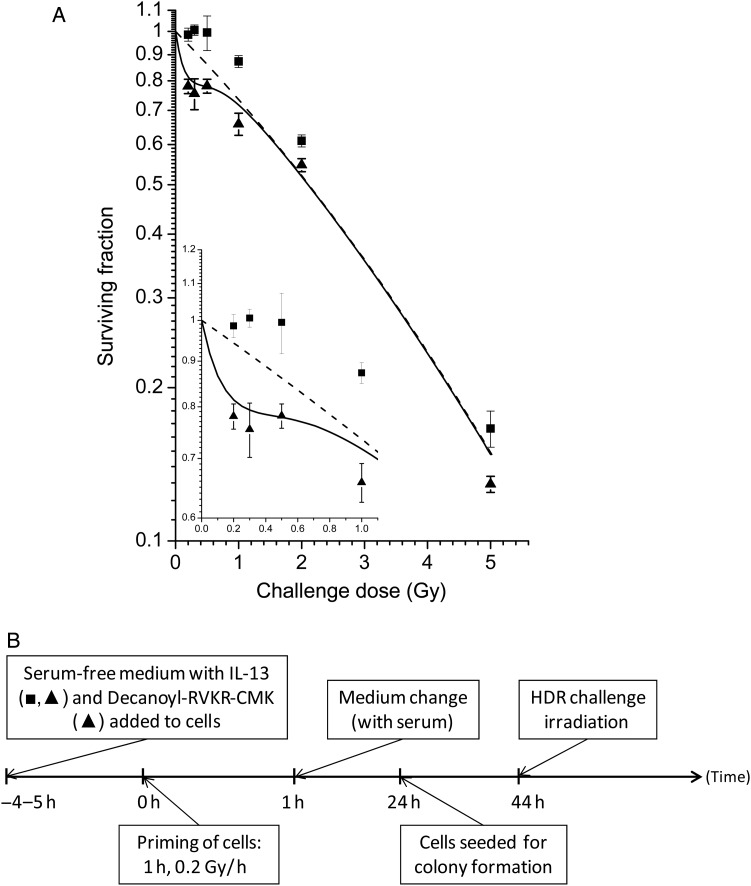
The response to HDR challenge irradiation in cells that were LDR primed in the presence of IL-13 as well as pro-protein convertase inhibitor Decanoyl-RVKR-CMK. **(A)** T47D cells were LDR irradiated in serum-free medium with10 ng/ml IL-13 and with (closed triangles) or without (replotted from Fig. 1A; closed squares) 20μM Decanoyl-RVKR-CMK, 44–20 h before HDR challenge irradiation (*P* = 0.05 for 0.5 and 2 Gy, *P* < 0.01 for all other doses). The curves represent model-fits to the data from unprimed cells by the IR-model (solid lines) and the LQ-model (dashed lines), respectively. The bars represent standard errors of the mean (SEM) for five individual experiments. **(B)** Timeline showing the experimental schedules.

TGF-β isoforms are synthesized as homodimeric proproteins (proTGF-β) that consist of TGF-β covalently bound to the latency-associated protein (LAP). The covalent binding is cleaved (by furin-type enzymes of the proprotein convertase family) as a proactivation, leaving TGF-β non-covalently associated with LAP in an inactive state. We hypothesized that IL-13 contributes to the proactivation of TGF-β3 by activating a proprotein convertase. Decanoyl-RVKR-CMK blocks the activity of all seven members of the proprotein convertase family. When this was added to the cell in serum-free medium together with IL-13, subsequent LDR priming did not remove HRS (Fig. 4). These results support the hypothesis that IL-13 activates one or more members of the proprotein convertase family.

**Fig. 5.**
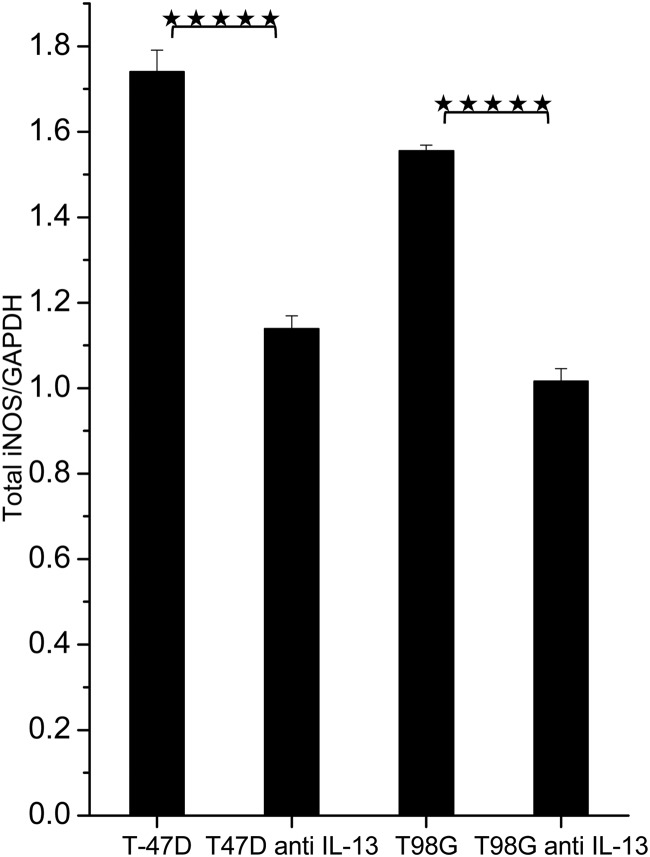
Total iNOS protein levels normalized to NAPDH levels using a cell-based ELISA assay. T-47D cells and T98G cells were treated overnight with neutralizing IL-13 antibody and compared with untreated cells. Five asterisks indicate *P* < 10^−5^. The bars represent standard errors of the mean (SEM) for three individual experiments, each with three samples.

We have previously shown that the effect of LDR irradiation on HRS is mediated by iNOS activation [9]. The requirement of IL-13 for LDR irradiation to have an effect might therefore be linked to iNOS upregulation. In order to test this, a cell-based ELISA assay was used to measure total iNOS levels in cells with and without IL-13 neutralizing antibody. For both T98G and T-47D cells, the iNOS levels were significantly reduced in cells exposed to IL-13 neutralizing antibody (Fig. 5). The relative amounts were 0.65 ± 0.02 for T98G cells (*P* = 4.7 × 10^−6^) and 0.65 ± 0.03 for T-47D cells (*P* = 6.3 × 10^−6^).

## DISCUSSION

The present study is to our knowledge the first to show involvement of IL-13 in the radiation response of cultured cells.

The requirement for fetal calf serum in medium for LDR irradiation to remove HRS indicated the presence of a factor in the blood of fetal calves that was not produced by our cultured cells. We hypothesized that this factor could be related to the immune system, and literature studies led to two possible candidates: IL-4 and IL-13. While IL-4 had no effect, adding IL-13 to serum-free medium restored the qualities of full serum medium with respect to the effects of LDR irradiation, indicating that IL-13 is the responsible factor in serum. The data from neutralizing IL-13 in full-serum medium (figure 3A) in combination with the data from adding IL-13 to serum free medium (figures 1 and 2) indicate that IL-13 alone may be responsible for the studied effect of serum.

Since IL-13 but not Il-4 binds to receptor IL-13Rα2, we tested inhibition of IL-13Rα2 and found that this receptor was indeed the mediator for the effect of IL-13. IL-13Rα2 has previously been seen to induce iNOS [15, 16]. In agreement with this, we found a reduced level of total iNOS protein in cells treated with IL-13-neutralizing antibody. We have previously shown that iNOS is activated in response to LDR irradiation and that peroxynitrite produced by superoxide and iNOS-generated NO is involved in mediating abolition of HRS [9]. This was the case both in the permanent effect of direct LDR irradiation of cells and in LDR irradiation of cell-conditioned medium resulting in a transient removal of HRS in recipient cells. Since the present data show that IL-13 is necessary for the effect of LDR irradiation in both cases, we hypothesized that the requirement for IL-13 in the medium for LDR irradiation to have the observed effect is in part due to a requirement for iNOS upregulation.

We have previously shown that TGF-β3 is activated by LDR irradiation and that TGF-β3 removes HRS [20]. The three TGF-β isoforms are all synthesized as homodimeric proproteins (proTGF-β) that have a mass of 75 kDa and consist TGF-β and LAP. The LAP is cleaved from the mature TGF-β 24-kDa dimer in the trans Golgi by furin-type enzymes as a proactivation, leaving TGF-β non-covalently associated with LAP in an inactive state [21]. In our previous study [20], we proposed that reactive oxygen species (ROS) produced by radiation can activate the proactivated non-covalently-associated TGF-β3, but that NO is required for scavenging LAP in order to prevent reassociation between TGF-β and LAP. Thus, it is likely that IL-13 is required to secure available iNOS to produce enough NO in response to LDR irradiation in order for LAP to be scavenged before reassociation to TGF-β3.

Liu *et al*. showed that receptor IL-13Rα2 is involved in the conversion of pro-furin to active furin [17]. Thus IL-13 could also be involved in the first step of proactivation of the TGF-β3-LAP complex. We were not able to detect any difference in furin levels in our cells using flow cytometry (data not shown). However, the supplier of the antibody (R&D systems, Minneapolis, MN, USA) could not exclude cross-reactivity with pro-furin. We therefore tried general inhibition of all seven members of the proprotein convertase family to which furin belongs and found that at least one of these was required for LDR priming to remove HRS. IL-13 thus appears to activate one or more enzymes of the furin family.

We propose that the active furin-like enzyme leads to proactivation of the TGFβ3-LAP complex as a prerequisite for TGF-β3 activation by LDR irradiation, which involves iNOS activation. The role of IL-13 appears thus to be two-sided: it proactivates TGF-β3 and facilitates activation by LDR irradiation by upregulation of iNOS.

In conclusion, we have shown that IL-13 is not produced by cultured cells but is present in the fetal calf serum used in cell culture. IL-13 interacts with the cells though receptor IL-13Rα2 and induces upregulation of iNOS and activation of one or more furin-like proprotein convertases.

## FUNDING

This work was supported by EU FP7 Grant No. 222741 (METOXIA), the Research Council of Norway, and the Norwegian Cancer Society. Funding to pay the Open Access publication charges for this article was provided by the University of Oslo.
